# Case report: Lung transplantation for treatment of paraquat intoxication: timing of transplantation

**DOI:** 10.3389/fphar.2023.1205689

**Published:** 2023-07-17

**Authors:** Congcong Li, Hongfei Cai, Fanyu Meng, Fanjie Meng, Ze Tang, Ying Tang, Ying Chen, Youbin Cui, Yang Li

**Affiliations:** ^1^ Department of Surgery, The First Hospital of Jilin University, Changchun, China; ^2^ Department of Thoracic Surgery, The First Hospital of Jilin University, Changchun, China; ^3^ Department of Respiratory and Critical Care Medicine, The First Hospital of Jilin University, Changchun, China; ^4^ Department of Critical Medicine, The First Hospital of Jilin University, Changchun, China; ^5^ Department of Thoracic Surgery, Organ Transplantation Center, The First Hospital of Jilin University, Changchun, China

**Keywords:** herbicide poisoning, lung transplantation, ECMO, bronchial infection, PLS

## Abstract

**Objective:** To analyze the optimal timing of lung transplantation and summarize postoperative complications and their management after paraquat poisoning.

**Methods:** Here, we present the clinical course of a 17-year-old boy with paraquat poisoning, in whom bilateral lung transplantation (LT) was performed. We reviewed the eight previously published articles relevant to LT after paraquat poisoning to summarize the therapeutic strategy.

**Results:** A 17-year-old boy was admitted to the hospital after ingestion of 30–50 mL 25% paraquat. Mechanical ventilation was initiated on the 25th day after intoxication. Venovenous extracorporeal membrane oxygenation was initiated on the 26th day. Sequential bilateral LT was performed on the 27th day. Several complex postoperative complications occurred and the patient was discharged on the 50th day postoperatively. Eight case reports were included in the literature review, including 11 patients with paraquat poisoning undergoing LT. Three patients died due to paraquat poisoning leading to fibrosis in the transplanted lungs or postoperative complications. Eight patients survived during follow-up.

**Conclusion:** LT after herbicide poisoning should be planned when hepatorenal function starts to recover, and waiting for complete recovery is unnecessary. Prevention of infection before surgery is important to reduce the incidence of postoperative infection. Complex perioperative complications caused by the herbicide itself or the late timing of transplantation can be successfully managed by a multidisciplinary team.

## 1 Introduction

Paraquat (1,1′-dimethyl-4,4′-bipyridine dichloride) is a contact herbicide that is widely used because of its low price, high efficiency, and ability of not being a pollutant of soil. Many people die from oral exposure accidently or for committing suicide, and there is practically no antidote for poisoning. An ingestion dose of 30 mg/kg or 50 mL of a 21% (w/w) solution may induce organ failure or death (Dasta, 1978). Poisoning could be fatal with doses as low as 1 mL (Teare, 1976); therefore, predicting the prognosis based on dosage is difficult. Only 1%–5% of paraquat is metabolized after ingestion. Gastrointestinal absorption occurs mainly in the small intestine, and unabsorbed paraquat is excreted through feces (Silva et al., 2015). The plasma paraquat concentration reaches its peak within 30 min to 4 h after absorption of paraquat, which is widely distributed in the body, spreading to various tissues and organs throughout the body (Dinis-Oliveira et al., 2008). The plasma paraquat is excreted almost unchanged in urine, exclusively by renal glomerular filtration and active renal tubular secretion within 12–24 h (Bismuth et al., 1987; Gawarammana and Buckley, 2011). Paraquat renal clearance is greater than creatinine clearance under normal renal function; however, renal tubular necrosis caused by paraquat results in the kidneys losing their ability to eliminate paraquat. Besides, reabsorption of paraquat through the proximal convoluted tubules is considered the reason for the inefficient renal clearance (Dinis-Oliveira et al., 2008). However, the incidence and severity of renal injury are lower than those of lung injury (Serra et al., 2003). Paraquat is strongly retained in the lungs early in the course (approximately 4 h after ingestion) owing to the involvement of the polyamine transport system; polyamines are abundantly expressed in the membranes of alveolar cell types I and II and Clara cells (Dinis-Oliveira et al., 2008). Polyamines and paraquat share a common uptake system; therefore, paraquat is mistakenly accumulated in the lungs, especially in alveolar type I and II cells and Clara cells (Dinis-Oliveira et al., 2008). After paraquat is taken in these cells, reactive oxygen species (ROS) subsequently generated with paraquat enter the redox cycling. ROS destroy alveolar epithelial cells, and extensive pulmonary fibrosis ensues, resulting dyspnea, cyanosis, and eventually death from respiratory failure (Walder et al., 1997; Jo et al., 2011; Shao and Chen, 2014; Ruaro et al., 2021). Current clinical treatments for paraquat poisoning include early gastric lavage, emesis induction, laxative, immunosuppressive agent, and mesenchymal stem cell administrations, and hemoperfusion. Although, these therapeutic measures are only supportive to slow the advancement of disease that is incurable in most cases. However, these treatments do not ensure the long-term survival of patients.

Lung transplantation (LT) was first reported to reverse lung fibrosis caused by paraquat in 1997 (Walder et al., 1997), and is the only effective treatment option for patients with respiratory failure and irreversible pulmonary fibrosis that continues to progress after paraquat poisoning. The timing of LT in paraquat poisoning is important for successful patient survival. Severe complications are often encountered by clinicians in the perioperative period. Here, we report the clinical course of recovery in a male student undergoing sequential double LT after paraquat poisoning, summarizing similar cases reported worldwide, to provide a reference for clinicians in future treatment.

## 2 Patients and methods

### 2.1 Study design and data collection

A17-year-old boy was scheduled to undergo LT for herbicide-induced lung fibrosis. We reviewed reported cases that documented detailed data and summarized the therapeutic strategy for transplantation timing and perioperative support. A summary of the cases is presented in Table 1. Continuous data with normal distributions are presented as medians with interquartile ranges.

**TABLE 1:** Cases summary.

Case report year	1968 (Matthew et al., 1968)	1973 (Cooke et al., 1973)	1985 (The Toronto Lung Transplant group, 1985)	1997 (Walder et al., 1997)	2014 (Jiao et al., 2021)	2015 (Tang et al., 2015)	2018 (Jiao et al., 2021)	2019 (Jiang et al., 2019)	2020 (Jiao et al., 2021)	2021 (Jiao et al., 2021)	2022 (Wu et al., 2022)
year/gender	15/male	18/male	31/male	17/male	24/female	21/female	45/male	26/female	38/male	30/male	18/female
poison volume (concentration)	1 moufful (20%)	1 moufful	exposure	NR (suicide)	50 mL (20%)	50 mL (20%)	60 mL (20%)	20 mL (20%)	50 mL (20%)	60 mL (20%)	NR (Be poisoned)
			10 years								
Preoperative liver function	dysfunction	dysfunction	NR	NR	stabilized	recovery	stabilized	NR	stabilized	stabilized	NR
Preoperative renal function	mild jaundice	oliguria	oliguria	oliguria	stabilized	recovery	stabilized	NR	stabilized	stabilized	NR
days to ECMO	-	-	14(R^a^), 24(L^b^)	44	44	44	38	35	27	28	34
ECMO mode	-	-	V-V^a^	CPB	V-V^a^	V-V^a^	V-V	V-V^a^&V-A	V-A	V-V	NR
days to LT	6	10	19(R^a^),41(L^b^)	44	56	56	38	58	27	28	34
LT type	left	single	right then left	left	bilateral	bilateral	bilateral	bilateral	bilateral	bilateral	bilateral
complications											
infection	no	NR	yes	no	yes	no	yes	yes	yes	yes	NR
bronchial stenosis/fistula	no	NR	yes	yes	no	no	yes	NR	no	yes	NR
respiratory stress/failure	yes	NR	yes	no	no	no	no	NR	no	no	NR
other	pneumothorax	NR	anuria	pneumothorax	no	no	thrombus	NR	no	no	NR
			myopathy	myopathy			pancreatitis				
outcome	death	death	death	survive	survive	survive	survive	survive	survive	survive	survive
survival time from toxicosis	19 days	12 days	110 days	>20 months	>5 years	>1year	>3 years	>1 year	>7months	>7months	>1 year
paraquat concentration											
serum (μg/L)	400 (day7)	NR	30 (day17)	0 (day4,18,50)	NR	248.96 (day46)	NR	NR	NR	NR	0 (day13)
			200 (day21)			30.53 (day56)					
urine (μg/L)	2,106 (day1)	NR	NR	0 (day 4,18,50)	248,960^b^	NR	229,200^b^	NR	148,600^b^	126,400^b^	70 (day13)
	0 (day17)										0 (day19)
tissue (μg/g)	removed lung 8.50 (day19)	NR	muscle (day21) 0.27 μg/mL	59th day: lung 134, muscle 328	NR	lung 0.38 (day56)	NR	NR	NR	NR	NR

NR, not reported; a, Right lung; b, Left lung.

^a^ ECMO, was instituted preoperatively.

^b^ At first admission, CPB, cardiopulmonary bypass.

### 2.2 Ethics

This study was approved by the Ethics Committee of the First Hospital of Jilin University. Written informed consent was obtained from the patient’s guardians. The transplanted organ was obtained from volunteer donation, and permission was obtained from the donor’s family. The Institutional Ethics Committee of the Organ Procurement Organization approved the donation procedure. Donor lungs were allocated through the China Organ Transplant Response System. The National Transplant Medical Review Board (Chinese Lung Transplantation Society and Transplantation Data Management and Quality Control Center) approved and registered the donor and recipient data.

## 3 Case presentation

**FIGURE 1:**
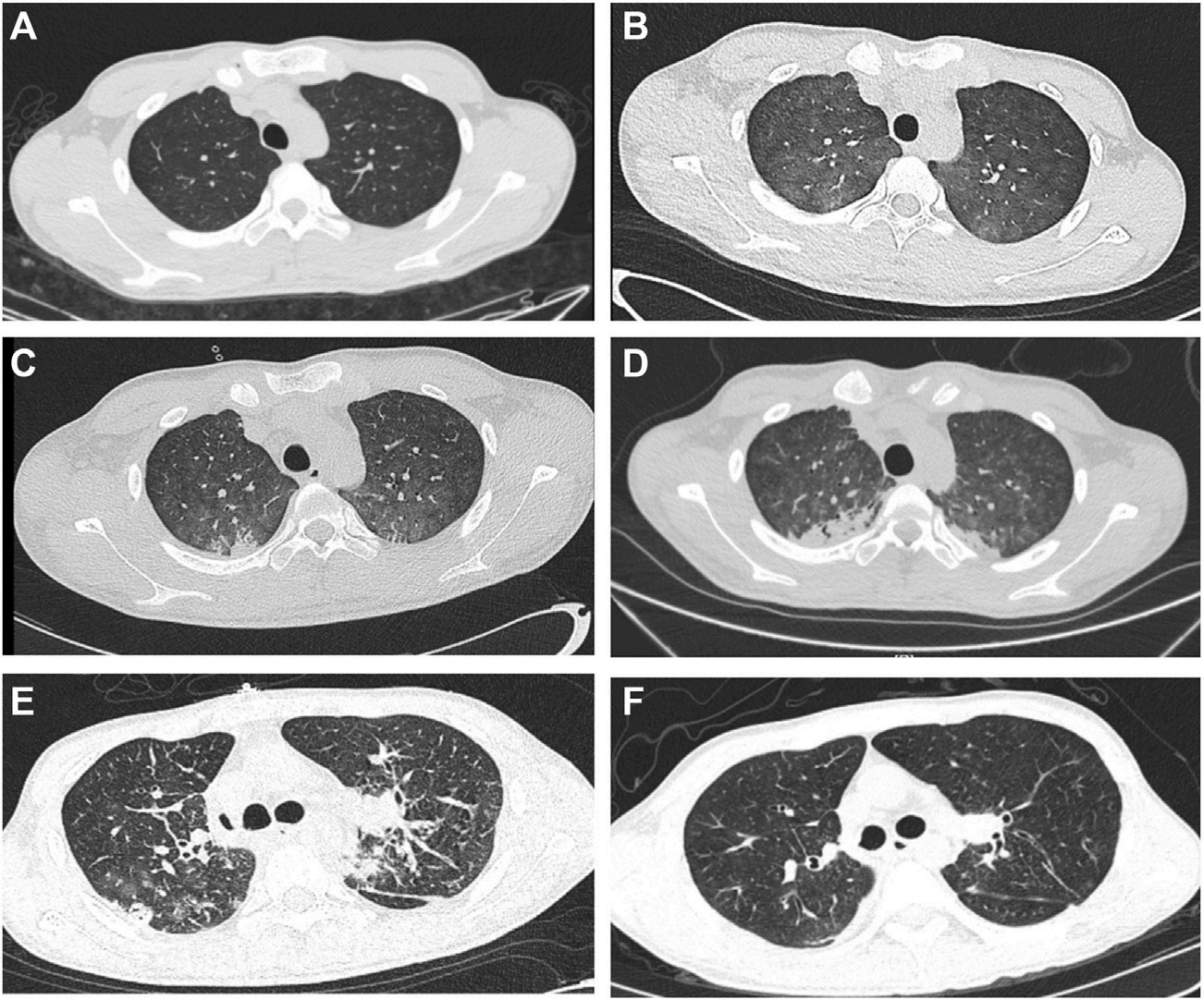
Preoperative CT images and postoperative CT images **(A–D)** Progression of bilateral lung fibrosis. **(E, F)** Post-lung transplantation CT shows normality and without fibrosis.

A 17-year-old boy ingested approximately 20–30 mL of 25% paraquat on 24 October 2022, and then developed nausea, vomiting, coughing, and general fatigue. He was transferred to a local hospital immediately after the poisoning, where he underwent gastric lavage, catharsis, and hemoperfusion twice. Multiple organ functions were damaged, and he was transferred to the First Hospital of Jilin University with these symptoms for advanced treatment on the second day of poisoning. The results of total computed tomography (CT), white cell count, hemoglobin level, albumin level were normal, but the creatinine level was 294.8 umol/L, blood urea level was 16.48 mmol/L, bilirubin level was 83.1 μmol/L, and serum alanine aminotransferase and aspartate aminotransferase levels were 450.7 U/L and 378.6 U/L, respectively. Dyspnea gradually worsened even with variable breathing support, and on the 25th day, the arterial blood gas analysis with inspired oxygen concentration of 100% showed oxygen tension of 43 mmHg and carbon dioxide tension of 87 mmHg. Therefore, mechanical ventilation was started to prolong life. Thoracic CT performed on 28 October 2022 (Figure 1B) demonstrated the progression of pulmonary fibrosis throughout the clinical course (Figure 1A–D). A multidisciplinary team of specialists evaluated the patient’s condition and agreed that LT was the only hope for survival. Extracorporeal membrane oxygenation (ECMO) was required to keep the patient alive to undergo LT. Venovenous ECMO (V-V ECMO) was performed on the 26th day of poisoning to increase the duration of survival for performing LT. On the 27th day, a lung donor with an O-positive blood group was found for our patient with a B-positive blood group. Bilateral sequential LT was performed through bilateral anterolateral incisions on November 20, with the patient on V-V ECMO support. Cold ischemia durations for the right and left lungs were 6 h 30 min and 7 h 30 min, respectively.

Postoperative immunosuppression was initiated with tacrolimus (FK506) and methylprednisolone; however, as the dosage of tacrolimus was adjusted, the blood drug concentration of FK506 decreased, failing to maintain the effective concentration. We then adjusted FK506 concentration with cyclosporine (CsA) dosage, and the blood drug concentration was terminally stable until posaconazole was added for a few more days.

Postoperative infection occurred, which was not surprising, by carbapenem-resistant *Acinetobacter* baumannii (CRAB). Vancomycin was administered for the initial 2 postoperative days, followed by a combination of meropenem, polymyxin B, and caspofungin. On the 3rd day of operation, the patient developed fever, for which minocycline combined with colistin sulfate inhalation was added. The treatment was changed to sulprozin combined with polymyxin E atomization and local spray under bronchoscopy after the patient’s condition stabilized. One day later, the CRAB disappeared.

Ten days after surgery, the patient’s hemoglobin level started decreasing from 130 g/L by an average of 20 g/L per day, without any symptoms. The diagnosis was challenging; then, passenger lymphocyte syndrome (PLS) was diagnosed. PLS occurs in patients with organ transplantation owing to an ABO blood type mismatch; antibodies produced from residual B lymphocytes transferred from the donor to recipient circulation against recipient’s red blood cell antigen cause hemolysis (Moosavi et al., 2020; Zhao et al., 2022). Fortunately, he patient’s condition gradually improved after the transfusion of washed red blood cells.

**FIGURE 2:**
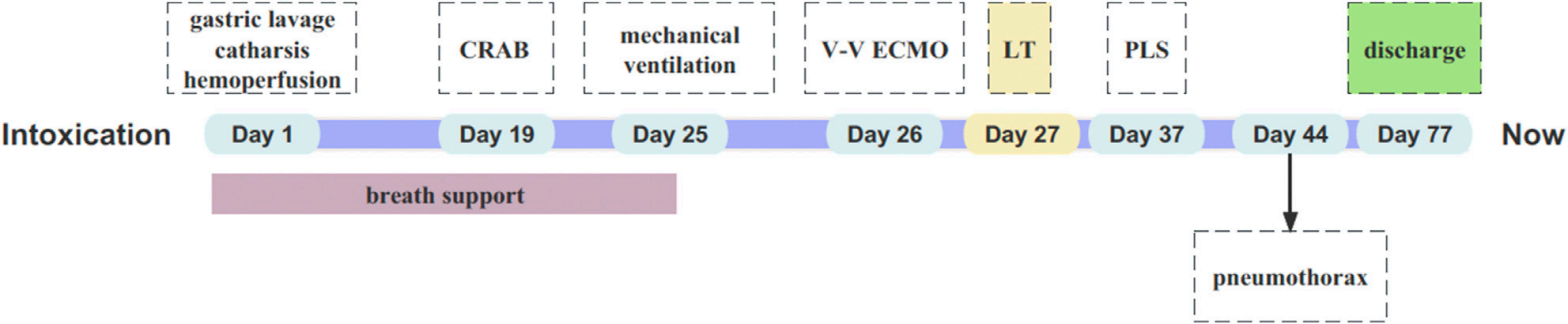
Timeline of clinical course in our case. CRAB: Carbapenem-Resistant *Acinetobacter* Baumannii; V-V ECMO: Venovenous ECMO; LT: Lung Transplantation; PLS: Passenger Lymphocyte Syndrom.

Rehabilitation training began after the patient was weaned off the ventilator and ECMO. The patient had poor pulmonary ventilation function, multiple muscle dysfunctions (respiratory, skeletal, and swallowing muscles), anxiety, and depression. Unfortunately, muscle biopsy was not performed to clarify the role of paraquat in causing the muscle weakness. Pulmonary function test results were as follows: forced expiratory volume (FEV_1_) 1.098 L and forced vital capacity (FVC) 1.323 L. The patient’s pulmonary function recovered to FEV1/FVC 82.9%, FEV_1_ 2.100 L, and FVC 2.820 L after muscle resistance training, psychological counseling, and drug therapy. Hyaline membrane-induced bronchial stenosis was observed, and cryosurgery, local polymyxin E spray, and periodic forceps removal under bronchoscopy were efficacious in our patient. The complications during hospitalization that included infection, PLS, pneumothorax, and emotional problems were well-treated with the efforts of our multidisciplinary medical team (Figure 2). We believe that the postoperative pneumothorax was caused by minor leakage at the anastomosis, because the anastomosis appeared normal on bronchoscopy. After continuous closed thoracic drainage, the pneumothorax was cured. The patient was discharged on the 50th day of surgery.

## 4 Results

We report the details of a 17-year-old boy who developed respiratory failure due to bilateral fibrotic lungs caused by paraquat intoxication who was resuscitated by LT. Details of the 11 previously reported patients are summarized in Table 1.

Before 1997, patients with pulmonary fibrosis in the end stage of paraquat poisoning who underwent LT died. The first case reported in 1968 was that of a 15-year-old boy who underwent single-left LT when his liver and renal functions deteriorated on the sixth day after ingestion of 1 mouthful of 20% paraquat who lived only for 2 weeks after the operation. Similar to the first case, the case of an 18-year-old man was reported in 1973, and he underwent single LT on the 10th day of intoxication in the presence of oliguria and died on the 12th day. The post-mortem analysis showed that the pathology of the transplanted lung was accordant with paraquat poisoning in the 2 cases. In 1985, a 31-year-old man who was exposed to paraquat spray and drenched clothes for over 10 years underwent right LT on the 19th day (from the appearance of the first symptom on 10 August 1985) and left LT on the 41st day with insufficient renal function. ECMO was initially instituted as a bridge to surgery as well as prolonged life support; he survived 110 days briefly and died because of a trachea-innominate artery fistula and progressive toxic myopathy. Successful survival of a patient with paraquat poisoning through single-left LT was first reported in 1997. A 17-year-old boy received LT on the 44th day after ingesting paraquat for suicide when renal function was restored. The boy in the 1997 case exhibited flaccid tetraplegia without sensory disturbance postoperatively, which were diagnosed using specific neuromyopathy through neuromyography and muscular biopsy. He received wedge resection and right pneumonectomy for a persisting leak on the 16th and 29th days, respectively, after left lung transplantation. Forty-seven days after operation, a bronchopleural fistula developed at the right inferior bronchial stump was occluded by local injection of aprotinin and thrombin-calcium chloride solution. Seven cases were reported in China from 2014 to 2022, and all patients received conservative therapy for a long period followed by bilateral LT with ECMO support after hepatorenal function stabilized. The median duration from poisoning to LT was 38 days (interquartile range 28, 56). Almost all patients had respiratory infections; fortunately, all survived. Furthermore, there was no evidence of re-fibrosis in the transplanted lungs in these seven cases.

## 5 Discussion

Numerous paraquat poisoning cases have been reported, mainly due to deliberate suicide, accidental ingestion, and chronic exposure. Paraquat is a corrosive bipyridyl compound, and its lethal concentration is incongruous (Dasta, 1978; Sukumar et al., 2019; Chandra et al., 2021). However, of the cases reported in 1973, a young boy took only one mouthful of paraquat and spat it out, but he eventually died after active treatment. Therefore, it is difficult to predict the patient prognosis based on the amount of paraquat ingested. Paraquat poisoning has a high mortality (50%–70%) (Sukumar et al., 2019) because of the unavailability of a detoxifying drug and the high treatment costs. Paraquat can be detected in the plasma as early as 30 min after oral administration, with the concentration peaking within 4 h (Senarathna et al., 2009; Liu et al., 2011; Cao et al., 2020). Mean distribution half-life and mean elimination half-life of the plasma paraquat concentrations are 5 hand 84 h, respectively (Walder et al., 1997).

Initiation of gastric decontamination *in situ* within the first hour of paraquat ingestion is essential (Elenga et al., 2018). The urine concentrations of four patients at their first admissions (2014, 2018, 2020, and 2021) are shown in Table 1. These were particularly highly in a case reported in 1968, in which 4.7 L of urine obtained by forced osmotic diuresis in the first 14 h after ingestion contained 99 mg of paraquat. Seventy percent of paraquat is eliminated by the kidneys, and different degrees of renal injury occurred in the cases reviewed. Early clearance (less than 4–5 h after the ingestion of paraquat), especially through hemoperfusion and hemodialysis, is associated with decreased mortality (Yen et al., 2018). Hemoperfusion and/or hemodialysis was performed immediately after swallowing paraquat in all surviving patients who underwent LT. The earlier the paraquat clearance treatment was performed, the more beneficial it was for patient survival.

Nausea, vomiting, oropharyngeal pain, and gastrointestinal symptoms develop immediately after paraquat ingestion. Clinical symptoms are characterized by gradually worsening hypoxemia in the later stages of poisoning and eventually develop into irreversible respiratory failure caused by progressive lung fibrosis. All cases reported after 2014 were treated with gastric lavage, hemoperfusion, hemodialysis, and administration of immunosuppressive agents, including methylprednisolone and cyclophosphamide, and antioxidant agents. The progression of bilateral pulmonary fibrosis did not stop or the condition did not improve even with ECMO support. LT is the only method to improve oxidation and increase the chances of survival of patients with paraquat poisoning, particularly as other visceral functions recover. There is no effective antidote for paraquat poisoning, the mostly empirical and supportive treatments before lung transplantation include emesis, gastric lavage, activated charcoal, laxative administration, purgation, and hemoperfusion/hemodialysis, antioxidants such as intravenous N-acetylcysteine and ascorbic acid, immunosuppressive agents and even mesenchymal stem cells. ECMO is crucial for bridging to lung transplantation.

All three reported cases of paraquat poisoning (1968, 1973, and 1985) that underwent LT surgery died. According to autopsy records, the reason for failure was not transplant surgery, but transplanted lung fibrosis. The duration from poisoning to receiving surgical treatment was short (6 days [1968], 10 days [1973], and 19 days [1985]). The hepatorenal function does not recover in a short time, which may be the main reason for their poor long-term survival. Since 2014, patients with paraquat poisoning have been reported to survive for LT, and the duration from poisoning to receiving surgery exceeded 1 month after hepatorenal function was stabilized or recovered. The duration of survival after LT intoxication was 27 days in our patient, and the creatinine and transaminase levels were not fully restored, but showed a downward trend. Owing to the presence of suitable donors, LT surgery could be performed urgently; therefore, complete recovery of hepatorenal function occurred in a short duration. Although the waiting time was short, transplanted lung re-fibrosis was not evident on postoperative CT images (Figure 1E, F). LT is feasible when hepatorenal function begins to recover and there is no need to wait for complete recovery. A prolonged waiting time can increase the risk of preoperative colonizing bacterial infections, which can result in complex airway complications that hinder recovery.

If the waiting time for LT is too short, the transplanted lung can exhibit fibrosis again, and if the waiting time is too long, the probability of airway complications such as preoperative infection and bacterial colonization is high. A more suitable transplantation timing needs to be explored. Surprisingly, we found the key time in the cases reported in 1968 and 2022: paraquat was not detected in urine on the 17th and 19th days, respectively. Although an accidental occurrence cannot be excluded, we believe that this may be a milestone in paraquat clearance. The time at which the drug cannot be detected in the urine is short, with an average of 18 days. Whether this time point is the optimal time for LT remains to be verified in further research.

However, plasma paraquat concentrations cannot be used to assess the extent of toxicant clearance from the body. In the case reported in 1997, paraquat was not detected in the serum at critical time points (fourth, 18th, and 50th days after poisoning). In the case reported in 1985, blood levels of paraquat increased by seven-fold after reaching a safe range (< 0.03 μg/L). In the case reported in 2015, the plasma concentration of paraquat was 30.53 μg/L on the 56th day of poisoning. Paraquat persisted in muscles and tissues for up to 2 months in these two cases. We hypothesized that remnants of paraquat in the serum originate from trace amounts released from the muscle tissue or other organs. Monitoring drug concentration in the serum after paraquat poisoning does not seem to have any decisive effect on the formulation of treatment strategies for patients.

Postoperative infection occurred in almost all patients with paraquat poisoning who survived LT. Postoperative infections seem inevitable due to multiple reasons, such as the long time from poisoning to LT, surgical trauma, weak respiratory resistance, and toxic damage. In our case, CRAB was evident in the sputum from the 19th day after intoxication until after the operation, when the leukocyte count exceeded 15 × 10^9^/L consistently. The patient was administered antibiotics *via* intravenous injection combined with atomization inhalation. Preoperative bacterial colonization in the airway poses considerable difficulties in postoperative anti-infection treatment. Proactive and targeted prevention of potentially pathogenic bacterial infections is important to improve prognosis.

Pneumothorax was diagnosed clinically by thoracic CT at the 30th day after operation in our patient, anastomotic dehiscence was not existed endoscopically. After thoracic drainage and bronchoscopic therapies including suction and spraying anti-infective drugs, the pneumothorax was cured. Bronchial stenosis is the most common airway complication following lung transplantation (Mahajan et al., 2017). Balloon dilation, forceps, and cryotherapy of proliferative necrotic tissue under bronchoscopy are effective (Orons et al., 2000; Chhajed et al., 2001; Shitrit et al., 2010; DiBardino et al., 2016). Bronchoscopic therapies has enhanced treatment options for lung transplant recipients with airway complications.

PLS occurs during organ transplantation in cases with an ABO blood type mismatch, where antibodies produced from residual B lymphocytes transferred from the donor to the recipient circulation against the recipient’s red blood cell antigens cause hemolysis (Moosavi et al., 2020; Zhao et al., 2022). Our patient suddenly exhibited a continuous decrease in hemoglobin levels during the stable recovery stage after surgery without any clinical symptoms. This led to the suspicion of occult blood loss or a hemolytic reaction during cross-matching. After PLS was diagnosed, washed red blood cells were transfused, leading to recovery. Fortunately, no severe hemolytic reaction complications such as respiratory distress, uremia, or liver failure occurred.

We were successful in rehabilitating a student with paraquat poisoning by involving a multidisciplinary team, including the Department of Mental Health and Rehabilitation. Several detoxification treatments and administration of immunosuppressive agents are effective and should be considered standard treatments in the future. The use of broad-spectrum antibiotics in the perioperative period is crucial for the treatment and prevention of infections. Tracheostomy plays a crucial role in the management of complex airways.

This report describes the case of a 17-year-old boy who ingested paraquat, underwent LT, and was discharged. LT after herbicide poisoning should be planned when hepatorenal function begins to recover, and waiting for complete recovery is unnecessary. Prevention of infection before surgery should be focused to reduce the incidence of postoperative infection. The complex perioperative complications caused by the herbicide itself or the late timing of transplantation can be successfully managed by a multidisciplinary team.

## 6 Conclusion

This report describes the case of a 17-year-old boy who ingested paraquat, underwent LT, and was finally was discharged. LT after herbicide poisoning should be planned when the hepatorenal function starts to recover; waiting for complete recovery is unnecessary. Prevention of infection is important before surgery to reduce the incidence of postoperative infection. The complex perioperative complications of the herbicide itself or late timing of transplantation can be successfully managed with a multidisciplinary team.

## Data Availability

The original contributions presented in the study are included in the article/Supplementary Material, further inquiries can be directed to the corresponding authors.
